# Creeping Through the Backdoor: Disruption in Medicine and Health

**DOI:** 10.3389/fphar.2020.00818

**Published:** 2020-06-10

**Authors:** Brendan Shaw, Orin Chisholm

**Affiliations:** ^1^Shawview Consulting, London, United Kingdom; ^2^Shawview Consulting, Sydney, NSW, Australia; ^3^Pharmaceutical Medicine, School of Medical Sciences, UNSW Sydney, Sydney, NSW, Australia

**Keywords:** disruption, healthcare, health systems, medicine, policy, innovation

## Abstract

Can disruption happen when no one notices? Disruptive technologies and processes are fundamentally starting to up-end how medicines and health systems benefit patients but the question is whether health systems are ready for them. This paper will briefly review the business strategy and management literature on topics such as disruption and “black swan” theories of change, before turning to discuss some of the areas where change is affecting medicine and healthcare. Such areas include the emergence of cell and gene therapies, the economics of cures, digital technologies, mobile apps, social media, supply chain technologies such as drones and online distribution, universal health coverage and funding, and consumerisation of healthcare. The question to be asked is whether these sorts of changes are “disruptive” or whether they were coming for a long time and it is just that health systems are slow to change. It could be argued that while perhaps unexpected by day-to-day practitioners in healthcare, in fact, many of the changes now starting to affect the health and medicines sector have been affecting other sectors such as technology, finance and communications for decades.

## Introduction

Even before the current total systemic disruption caused by the Covid-19 outbreak, health systems worldwide were increasingly confronted with immense change and disruption. But what does “disruption” actually mean and do health systems cope well with disruption? In fact, health systems are not well suited to dealing with disruptive change (Morris et al., 2011; Chaudoir et al., 2013) but they are not unique in this problem (Rogers, 2003). Much of the literature on disruption from the world of business is filled with examples of organizations that do not adapt to disruptive change very well (Wright et al., 2004; Burke, 2018). Given the scale of change sweeping society today, it is important to look at how adaptable our health systems are to change now and in the future.

## What Is Disruption?

Innovation and disruption has become a major theme in business studies and management research. The term has been adopted across a range of fields to describe radical change that disrupts current paradigms. Stemming from earlier work by economists such as Joseph Schumpeter’s concept of “creative destruction,” Clayton Christensen has examined how disruptive innovation affects businesses and industrial sectors (Schumpeter, 1942; Spencer and Kirchhoff, 2006; Christensen et al., 2017).

While the term disruptive innovation is used at length, one problem is that the term has not been sufficiently defined in a number of disciplines. While scientific research, technological change, business development and other forms of innovation occur all the time, innovation that can be considered “disruptive” is that which up-ends the established order or way of doing things. In the context of healthcare, Christensen has defined disruption as “an innovation that makes things simpler and more affordable, and ‘technology’ is a way of combining inputs of materials, components, information, labor, and energy into outputs of greater value” (Christensen et al., 2017). However, this definition has been criticized because it fails to sufficiently differentiate what is disruptive and what is not (Weeks, 2015). Moreover, the experience in healthcare is that innovation does not always lead to more affordable technologies.

At one level innovation is part of the normal operation of health systems. Scientific research is constantly leading to more knowledge about health and disease; new medicines and medical devices are being developed; and new surgical and medical procedures are being incorporated into healthcare. Some of this innovation will provide substantial “breakthrough” technological improvements, some will be incremental improvements and some will provide little or no benefit. Evaluation systems like health technology assessment are often used to assess their benefit and cost-effectiveness for the health system (Caro et al., 2019).

Our definition is that disruption is where a change upsets current systems to such a point that the incumbents operating in that system cannot adapt to that change. Innovation and change happen all the time, and health systems are no exception to this. Our interest here is in how disruption upsets current systems, upends existing paradigms and allows outside entrants to enter the market and introduce substantive innovations, models or processes that radically shift the dynamics and accepted processes in a system that are not well anticipated or managed by the incumbents in a system.

## Theories of Disruption

A key learning from the management literature is that organizations often find it difficult to embrace radical, disruptive innovations in their own business strategies (Wright et al., 2004; Burke, 2018). This can be due to companies’ existing customers not wanting disruption and preferring a companies’ older products, processes and technologies (Christensen et al., 2015). This provides companies with the challenge of introducing innovations to attract new customers while maintaining their existing business and customer base (Markides and Oyon, 2010). Companies themselves might be reluctant to disrupt their existing profits, business models and processes that might still be earning good returns for the company today. Existing company frameworks and belief systems might also hold back the adoption of new processes and technologies (De Jong and Van Dijk, 2015).

While not necessarily being a forgone conclusion, there are numerous examples of companies that failed to disrupt themselves in the face of emerging technological and business changes leaving the way open for competitors to take advantage (Ismael et al., 2014; Gans, 2016). Kodak and its failure to capitalize on its invention of the digital camera (Mui, 2012), Nokia’s failure to adapt to the mobile phone market (Birkinshaw, 2013), and Xerox failing to succeed in personal computers despite developing many of the core technologies (Wessel, 2012) are examples of this.

Uncertainty is also a problem, where a company or organization may have a range of potential technological options available but it is unclear which is the best choice. Decisions about the future must be made in the absence of perfect information (Nooraie, 2012), a problem complicated by the increasing pace of disruption compressing the timeframes available to embrace radical changes (Baghai et al., 2000; Scott et al., 2018). “Black swan theory” in the management literature explores the extent to which organizations and societies are ready to manage large or highly impactful events that are outside the bounds of what people would consider ‘normal’ (Taleb, 2007). Applied to risk management and decision making in business, this theory encourages companies to think outside their normal frame of reference in considering future scenarios (Le Merle, 2011; Ferguson, 2014).

Disruption is increasingly becoming a normal part of business strategic planning. This is triggered by a swathe of enabling technologies across sectors and novel business models introduced by new entrants with first-mover advantages over incumbent firms (Spencer and Kirchhoff, 2006). Examples include Uber in the hire taxi car business, Netflix in the on-demand television subscription market, Tesla in the automotive industry, and Amazon in the online and retail shopping sector (Voigt et al., 2017; Berger et al., 2018; Ives et al., 2019; Shipley, 2020).

## What Disruptions Are Occurring in the Health System?

Our sense is that, after a slow start, disruptive change is starting to affect health systems around the world in many ways. Some of the changes now starting to impact the health sector have been disrupting other sectors in society for some time, but are now gathering momentum in the health sector: Artificial Intelligence (AI)/machine learning, quantum computing, blockchain, robotics and the Internet of Things (expect acceleration with the roll-out of 5G networks). There are suggestions that the pace of disruption will increase in healthcare as in other sectors of the economy and society. This may be due to fundamental structural changes including technological change in things like nanotechnology, quantum computing and robotics, or changes in consumer behavior, such as a greater focus on self-help, access to information and social connection (Deloitte Center for Health Solutions, 2019).

### Technological Changes

Many of the changes disrupting healthcare stem from technological change. For example, new cell and gene therapies which offer potential cures but also raise challenges for regulatory agencies and payers (Schaffer et al., 2018; Anguela and High, 2019; Faulkner et al., 2019; Jonsson et al., 2019). The growth in AI, machine learning and big data in healthcare has the potential to disrupt the science and economics of drug development and healthcare in various ways (Harrer et al., 2019; Topol, 2019; Joshi et al., 2020; World Health Organisation, 2020). Examples include tracking population outcomes, tracking health epidemics such as thunderstorm asthma and the Covid-19 outbreak or improving clinical trials and drug development. The recent publication of Deep Mind’s AI predicting the shape of proteins faster than humans (Senior et al., 2019), the commencement of clinical trials for a new influenza vaccine developed using AI (Masige, 2019) or the use of machine neural learning networks to identify new antibiotics or optimize orthopedic surgery (see **Box 1**) show the potential of this technology. Internet-based technologies such as blockchain are predicted to provide opportunities for health system functionality and efficiency by developing new payment mechanisms, improving the integrity of medical records and upgrading supply-chain integrity (Halamka et al., 2019). 3D-printed pills have already received FDA approval (FDA, 2015; Stokel-Walker, 2019; Tangermann, 2019) and, as they become more mainstream, may disrupt traditional delivery and supply systems of medicines. Novel drug-device combinations are entering the market such as Abilify MyCite (aripiprazole pill with ingestible event marker sensor to track compliance), approved by the FDA in 2017 (Otsuka Pharmaceuticals, 2017). This will disrupt tracking of compliance in certain patient populations (Velligan et al., 2019).

Box 1 **Examples of Disruptive Technological Changes in Healthcare.**

**AI in Drug Discovery**

The latest deep neural machine learning networks in artificial intelligence have been used to identify a new antibiotic, halicin, which has the potential to treat many drug-resistant bacteria. Researchers have demonstrated the ability to use such processes to rapidly search through millions of potential candidate molecules. Halicin is a molecule identified and recommended by machine learning and was found to be effective against many drug-resistant bacteria such as *Mycobacterium tuberculosis* and carbapenem-resistant Enterobacteriaceae. As well as identifying a potential new antibiotic molecule to help address a major worldwide health problem in drug-resistant bacteria, the new technology also demonstrated the benefits of the application of artificial intelligence to drug discovery. The use of machine learning could rapidly increase the rate at which new medicines are discovered in ways much faster than humans could, reduce the time needed to screen potential new treatments and reduce the costs of drug development (Stokes et al., 2020).

**AI in Assisted Surgery**

360 Knee Systems is focussed on improving outcomes for patients needing total knee replacements (https://kneesystems.com/). Their disruptive system is based on pre-operative patient profiling, scanning the patient’s knee and building a computer model to optimize placement of the 3D-printed knee. They utilize cameras in the operating theater coupled with real-time feedback to the surgeon to ensure accurate placement for optimal patient movement post-surgery. The current model of treatment relies on the expertise of the orthopedic surgeon, often resulting in an unacceptable revision rate. This AI-assisted method would decrease the revision rate, reducing overall costs to the healthcare system. Recovery is managed by a patient-specific rehabilitation program delivered both in-person and by telemedicine, again utilizing disruptive technology to improve patient care.

### Economic Changes

Systems of health technology assessment may be forced to reconsider conventional value assessment and reward models because health system management has not anticipated the impact of the rapid evolution of breakthrough therapies (Faulkner et al., 2019). While the latest generation of hepatitis C medicines were evaluated as cost-effective, some governments had difficulties in structuring their policies, diagnostic and financing systems to manage the budgetary impact (World Health Organisation, 2018). New payment models such as subscription-based ‘Netflix’-type models and others have been proposed (Moon and Erickson, 2019; The Economist, 2019).

The potential for rapid disruption in the supply chains for medicines, blood supply and other health services has increased with the entry of disruptors from other sectors outside healthcare such as Amazon’s purchase of PillPack (Baum, 2018), Google buying FitBit (Berg, 2019; Lovett and Muoio, 2019) or Alphabet’s drug development partnerships (GSK, 2016; Pharma Letter, 2019). Rapid development and falling costs in drone technology means that these are increasingly being ulitised to deliver healthcare products and services (Amukele, 2020; Ochieng et al., 2020), be it the delivery of vaccines to remote health clinics in Vanuatu, blood supplies in Rwanda, or insulin to remote towns in Ireland, disrupting traditional supply chains (The Lancet Haematology, 2017; McVeigh, 2018; McGrath, 2019; Poljak and Šterbenc, 2019). Thermostable vaccines will also disrupt supply chain models (Lee et al., 2017). Such technologies, along with the rise in innovative models for public-private partnerships will deliver benefits to public health (National Academies, 2019). We are already starting to see this in the Covid-19 pandemic (Mullin, 2020).

### Societal Changes

Societal and environmental disruption is affecting healthcare. For example, the growing use of social media by patients and doctors alike (George et al., 2013) has potential benefits through improved patient information and public health research. However, social media also carries significant risks through the spread of dis-information such as the rise of anti-vaccination groups; the recent Samoan measles outbreak being a case in point (McNamara, 2019). A call from the Director-General of the World Health Organization (WHO) for a collective effort to combat the promotion of misinformation about vaccines through social media signals how problematic this disruption has become for healthcare (WHO, 2019a). Similarly, climate change is listed by the WHO as one of the top ten threats to human health in 2019 (WHO, 2019b). There is growing recognition that climate change is creating major challenges for healthcare systems, be it through the rise of mosquito-borne infectious tropical diseases in countries where these have historically been absent (Franklinos et al., 2019), such as the rise of West Nile virus in Germany (Wyatt, 2019), or the rise of malnutrition and heat stress (World Medical Association, 2019).

Covid-19 has accelerated the roll-out of services that reduce face-to-face interactions in healthcare. For example, video consultations between doctors and patients have been rapidly implemented in Australia, over a couple of weeks (Greenhalgh et al., 2020); see **Box 2** for further examples.

Box 2 **Addendum—Covid-19.**

At the time of this article going to press, the Covid-19 outbreak is taking hold in many countries around the world, putting many health systems under enormous pressure. As an example of disruption in healthcare, it is perhaps one of the most fundamental disruptive challenges for health systems in over a century. It might be argued that the Covid-19 outbreak is not an example of disruption creeping through the backdoor and might therefore rebut some of the arguments made here about health systems’ latent difficulties in adopting disruptive technologies. However, we argue that the Covid-19 outbreak supports the essential arguments made in this article. One reason being the numerous warnings made by health experts in the years leading up to the outbreak about the fact that health systems needed to be better prepared for a likely future pandemic, including coronaviruses as a possible cause (Osterholm, 2005; Cheng et al., 2007; World Health Organisation, 2016; World Health Organisation, 2019b). Moreover, many of the technologies now being hastily introduced into health systems in response to the Covid-19 outbreak, such as widespread use of telemedicine in doctor-patient consultations (Webster, 2020), drone deliveries of medicines to communities (Hawkins, 2020) and local manufacturing of protective equipment using 3D printing (Philipkoski, 2020), are all technologies that existed and were talked about in the context of healthcare. However, in these instances it has taken a global catastrophe like Covid-19 to nudge health systems around the world to more systematically implement some of these technologies. Finally, there is a need to undertake further research into the impact of the Covid-19 outbreak and how health systems dealt with such fundamental disruptive shocks.

## Can Health Systems Cope With Disruptive Innovation?

We believe that health systems have great difficulty adapting to disruptive innovation. The United Kingdom’s National Health Service (NHS), for example, “does not have a strong track record” in implementing new technology (Castle-Clark, 2018). It still purchases 10% of the world’s pagers (Guardian, 2017; Gulland, 2017) and is the largest purchaser of fax machines in the world (Rose, 2017; Bodkin, 2018). Business analysts have noted health systems’ slow response to change: introducing AI into healthcare is like ‘grafting Star Trek on to the Flintstones’ (Murphy and Nirad, 2018; Dearment, 2019; Kirsch, 2019). The Organization for Economic Co-operation and Development (OECD) has also suggested that health systems do not evolve rapidly (OECD, 2019).

One reason change can be difficult is that political issues, often embedded in healthcare reform, can stifle or restrain even gradual changes in policy. This reluctance may exist in governments, payers, regulators, healthcare professionals or incumbent private companies. Institutional and political structures in health systems can dictate the extent to which rapid, disruptive changes are embraced by health systems (Stuckler et al., 2010; Sparkes et al., 2019). Governance and policy have not kept pace with disruptive challenges facing health systems, including things like digital technological developments (Kickbusch et al., 2019; Shaw and Chisholm, 2019). Rather like the companies in other industries that never quite implement disruptive innovation because of their own internal incentives and structures, similarly in healthcare these same legacy issues can apply (Christensen et al., 2017).

One example of this is the emergence of cell and gene therapies which are now upending established medical practice, regulatory systems, reimbursement and purchasing models (Institute for Clinical and Economic Review, 2018; Hay and Cheung, 2019). The human genome was sequenced at the turn of the century and even then presidents and prime ministers spoke about this milestone leading to genetic-level treatments for disease (CNN, 2000). So, why weren’t health systems ready for their arrival?

While some developments might be viewed as “black swan” moments, perhaps such changes could have been anticipated if existing mindsets and frames of reference had been challenged. How many disruptive changes are truly surprises or just hidden in plain sight?

## Implications for Future Healthcare and Health Policy

The problem for decision-makers in healthcare, be they governments, payers, regulators, companies, healthcare professionals, or patients, is that recognizing disruptive innovation when it is occurring can be difficult. The business literature tells us that the reasons companies find it difficult to see disruptive innovation coming is because it is uncertain, information may be incomplete and the innovation initially appears inoccuous, ineffective or inefficient. In such an environment it can be difficult to see disruptive innovation emerging or difficult to do anything about it when it can be seen. “Successful companies make conscious decisions to deemphasize or seemingly ignore those innovations until it is too late. They are stuck in an unenviable dilemma that prevents them from moving toward the new for fear of losing too much of the old” (Gans, 2016, p. 84).

One of the biggest drivers of disruption in healthcare is patients, demanding the same level of innovation, service and quality from health service providers that they see in other service sectors like online shopping, travel, and media (Murphy and Nirad, 2018). Patients and the broader community are increasingly demanding better service and care, either through their wallets or the ballot box.

The role of the private sector in healthcare will become more, not less, contentious. Already a major topic of debate in the provision and rollout of universal health coverage (Akinola and Dimitrova, 2019; Clarke et al., 2019), given that in other sectors of the economy much of the disruptive innovation has been driven by the private sector (telecommunications, IT, media and travel). What role can and should the private sector have in transforming healthcare in the future? And which private sector should it be? The traditional private sector players in healthcare, such as pharmaceutical companies, private hospitals, insurance companies and private medical practices may find themselves increasingly challenged by outsiders like Google, Amazon and Uber. Will patients and public policy makers welcome or push back against the entry of new private sector players in healthcare?

Just as in other sectors of the economy where organizations have had to adapt to change, actors in the health sector will need to improve their adaptability to change. Governments will need to become more adept at managing regulatory and policy challenges that open up new opportunities. Payers will be confronted with radical new medical treatments that challenge traditional financing systems but save money over the long-term. Pharmaceutical and device companies will be threatened by new entrants into healthcare from outside the sector but could also build new business. Healthcare professionals may be challenged by new technologies that undermine old treatment pathways but create new ones and patients may be confronted by new tests or data that seem threatening but deliver greater quality of life.

How and why health systems manage such disruption will vary depending on factors such as the level of public versus private funding, the relative roles of different actors in the sector, the degree of institutionalization and legacy in the system, the relative level of resources in a country, the extent to which a country’s health system is internationalized and population characteristics and needs.

Several areas of further research emerge from this overview. Firstly, a clearer definition of disruptive innovation is needed, particularly in the healthcare setting. While various clarifications have been suggested the ongoing debate about what disruptive innovation actually is suggests further refinement is needed around this definition (Hwang and Christensen, 2008; Kirsch, 2019). Moreover, further research to understand what types of health systems are best able to cope with, manage and utilize disruptive innovation would be beneficial (Hwang and Christensen, 2008; Broska et al., 2019; Deloitte Center for Health Solutions, 2019; Gaddam, 2019).

## Conclusions

As the current Covid-19 outbreak is graphically demonstrating, disruption in health often happens in spite of, not because of, the structures and processes of health systems. Disruptive innovation provides both challenges and opportunities in healthcare. While raising many issues that are, at times, confrontational for health systems, disruption can cause problems but also provide new solutions. The challenge is in being able to adapt, encourage, and respond to such change.

Many of the disruptive forces now starting to affect health systems have been building for some time, as evidenced by their earlier emergence in other sectors of the economy and society and such changes will continue to trigger rapid and dramatic changes in healthcare.

All players in the health system, be they governments, payers, patients, healthcare professionals, providers and technology developers have valid roles in managing and adopting disruptive changes in healthcare, as demonstrated by their responses to the recent Covid-19 outbreak. The challenge is for those players in the health sector to recognize and capitalize on disruptive changes to the benefit of patients and the community worldwide while delivering a more efficient and effective health system for the 21^st^ century and beyond. While potentially generating enormous opportunities for society, including better health, social development and economic growth, health systems and the participants in it need to be ready to anticipate, consider and embrace such changes as they come through the backdoor.

## Author Contributions

BS and OC were responsible for the concept and writing of this paper and take full accountability for the content.

## Conflict of Interest

BS carries out research and consulting projects for pharmaceutical firms and other public, private, charitable and community organisations.

The remaining author declares that the research was conducted in the absence of any commercial or financial relationships that could be constructed as a potential conflict of interest.
